# Changes in Ergosterol Biosynthesis Alter the Response to Cycloheximide, 4-Nitroquinoline-N-Oxide, Weak Organic Acids, and Virulence in *Candida glabrata*

**DOI:** 10.3390/jof10100669

**Published:** 2024-09-26

**Authors:** Daniel Eliaš, Nora Tóth Hervay, Lucia Černáková, Yvetta Gbelská

**Affiliations:** Department of Microbiology and Virology, Faculty of Natural Sciences, Comenius University in Bratislava, Ilkovicova 6, 842 15 Bratislava, Slovakia

**Keywords:** *Candida glabrata*, *ERG6*, *Galleria mellonella*, rhodamine 6G, virulence, ergosterol

## Abstract

The *ERG6* gene encodes the sterol C24-methyltransferase converting zymosterol to fecosterol in the ergosterol biosynthetic pathway. Here, we extend the results of functional analysis of the *CgERG6* gene, which was previously shown to modulate drug susceptibility in *Candida glabrata* mutant cells, by demonstrating that its deletion leads to increased susceptibility to cycloheximide, 4-nitroquinoline-N-oxide and weak organic acids, and such effects are associated with attenuated virulence. Together with abrogated efflux of drug substrates by *Cg*Cdr1p and *Cg*Pdr12p, the *Cgerg6Δ* mutation leads to reduced cell surface hydrophobicity and decreased virulence of the mutant cells of *C. glabrata*. The absence of *Cg*Erg6p impacts the lipid organization and function of the plasma membrane, resulting in non-specific permeability and abrogation of normal function of membrane-bound proteins accompanied by decreased virulence in *Cgerg6Δ* cells. *Galleria mellonella* larvae were used as a non-vertebrate animal host model to determine differences in the virulence potential of *C. glabrata* strains (parental strain and the *Cgerg6Δ* deletion mutant). We found that *Cgerg6Δ* mutant strain attenuated in virulence caused 25–30% survival of larvae compared with parental strain.

## 1. Introduction

*Candida* spp. are the most common fungal pathogens affecting humans. *Candida albicans* and *Candida glabrata* account for 70 to 80% of the yeasts isolated from patients with invasive candidiasis. Three classes of antifungal agents—azoles, polyenes, and echinocandins—are approved for the treatment of candidemia and candidiasis [1,2,3]. However, an important factor that can cause therapeutic failure is the infecting *Candida* yeast species’ antifungal drug resistance to antimycotics. *C. glabrata* is important because of its rapidly developing resistance to currently available antifungals, mainly the azoles [4]. Resistance to azoles is caused by increased drug efflux, alterations in the target binding site of the drug, or changes in the biosynthetic pathways that circumvent or weaken the effect of the antifungal drug used [5]. In *C. glabrata*, overexpression of the ABC efflux pumps *CgCDR1*, *CgPDH1* (*CgCDR2*), and *CgSNQ2*, as well as the MFS transporters *CgFLR1* and *CgQDR2*, has been shown to contribute to azole resistance in clinical isolates [6,7,8,9]. Gain-of-function (GOF) mutations in the transcription factor *Cg*Pdr1p induce the expression of ABC efflux pumps and the MFS transporter *CgQDR2* [10,11]. GOF mutations in *CgPDR1* also regulate virulence and lead to increased expression of adhesin proteins, such as Epa1p, resulting in increased adherence [12]. In addition, mutants of *C. glabrata* with non-functional mitochondria are azole-resistant, which correlates with the upregulation of genes for efflux pumps, etc. [6].

A common target of antifungal polyenes and azoles is the fungal ergosterol and its biosynthetic pathway. Ergosterol is the predominant fungal sterol, which is not found in mammalian cells, that produces cholesterol. Because of this difference, fungal ergosterol and its biosynthesis are successful targets for the treatment of fungal infections in humans, animals, and plants with antifungal drugs. Ergosterol biosynthesis and its regulation is conserved in eukaryotes and has been studied in most detail in the yeast model of *Saccharomyces cerevisiae* [13,14,15,16]. The single deletion of most genes for ergosterol biosynthesis is lethal under standard growth conditions without the addition of ergosterol. The only exceptions are the last five enzymes, which are encoded by the *ERG2* to *ERG6* genes, probably due to the similar physiochemical properties exhibited by the intermediates accumulated in these mutants with respect to ergosterol and *ERG28*. These *ergΔ* mutants, which either lack sterols or produce alternative sterols, exhibit a range of phenotypes and show alterations in stress resistance [17,18]. Azoles affect Erg11p by binding directly to the iron atom in the heme group of the enzyme [19]. When Erg11p is inhibited, an alternate pathway is activated, leading to the formation of the toxic, fungistatic 14α methylergosta 8–24(28) dienol [20,21]. Mutations in the *ERG6* and *ERG3* genes, which are involved in this alternative pathway, lead to the development of azole resistance [21,22]. Polyenes, including nystatin and amphotericin B, interact directly with ergosterol on the cell surface [23]. The *ergΔ* mutants studied to date, deletion, and overexpression of the *ERG6* gene encoding the sterol C24-methyltransferase result in the most severely impaired phenotypes, suggesting that it may be a suitable target for a new generation of antifungal agents [21,24].

In the context of emerging resistance, the development of antifungals with novel mechanisms of action against a fungal-specific metabolic pathway and/or potentiation of the efficacy of current antifungals is a priority for the research community. In addition to exploiting subtle structural and functional differences in metabolic enzymes that are common to both fungi and humans, intracellular redox and environmental regulators, as well as key phosphatases, kinases, and transcription factors that form cellular signaling networks have been explored as potential drug targets. In the last decade, new anti-*Candida* drugs have been tested in clinical trials based on different binding sites of the same target or on completely new targets, as well as on improved formulations of existing drugs [25,26].

In our previous work [27,28] we have shown that the absence of the sterol C24-methyltransferase, which converts zymosterol to fecosterol in the ergosterol biosynthetic pathway, leads to alterations in the susceptibility of *C. glabrata* to various antifungal agents and plasma membrane properties. This study aims to investigate the role of *CgERG6* in the virulence and drug resistance of *C. glabrata* by analyzing the phenotypic consequences of its deletion.

## 2. Materials and Methods

### 2.1. Yeast Strains and Media

The *Candida glabrata* strains used in this study were *Cglig4Δ lig4::HIS3trp1* [29] (in further text appointed as parental strain) and its isogenic derivative *Cglig4Δ erg6Δlig4::HIS3erg6::TRP1* [27] (appointed as *Cgerg6Δ*). The *Cglig4Δ* deletion strain was kindly provided by Patrick van Dijck (KU Leuven, Leuven, Belgium). *C. glabrata* cells were cultivated in YPD medium (1% yeast extract, 2% peptone, 2% glucose). For solid media, 2 g per 100 mL of agar was added to the liquid medium mentioned.

### 2.2. Susceptibility Assays

The susceptibility of *C. glabrata* strains to various chemicals was tested by spot assays. Overnight yeast cultures were grown in YPD medium diluted to a density of 1 × 10^7^ cells/mL. Tenfold serial dilutions were prepared and 5 μL aliquots of each dilution were spotted onto solid YPD plates, containing the indicated concentrations of the tested compounds. The drug concentrations used were as follows: cycloheximide: 0.5, 1, 1.5, 2 μg/mL, 4-nitroquinoline-N-oxide (4-NQO): 1, 2.5, 5 μg/mL, benzoic acid: 5, 7.5, 10, 15, 20 mM, propionic acid: 5, 7.5, 10, 15, 20 mM, sorbic acid: 5, 7.5, 10, 15, 20 mM. Colony growth was scored after 48 h at 30 °C. The experiments were repeated five times. The susceptibility of strains to rhodamine 6G was assessed by disk diffusion. Yeast cultures adjusted to 1 × 10^7^ cells/mL were spread onto YPD solid media. Sterile filter papers containing 5 mM and 20 mM of rhodamine 6G (Sigma-Aldrich, Taufkirchen, Germany) were placed onto the surface of YPD media. Diameters of growth inhibition zones were measured after 24 h at 30 °C. The disk diameter was 5 mm. The experiments were repeated three times.

### 2.3. Rhodamine 6G Accumulation and Efflux

Accumulation of rhodamine 6G was determined as previously described [30]. Briefly, overnight cultures were resuspended in 100 mL of fresh YPD medium to concentrations of 5 × 10^6^ cells/mL and grown at 150 rpm, 30 °C to 1 × 10^7^ cells/mL. Yeast cells were washed with sterile deionized water and HEPES buffer (50 mM, pH 7.0). Cell suspensions were subsequently resuspended in 10 mL of HEPES buffer with 2 mM 2-deoxyglucose, and a final concentration of 10 µM of rhodamine 6G was added to the cell suspension incubated at 30 °C. Incubation of yeast cells in the presence of 2-deoxy-D-glucose (inhibitor of glycolysis) and Rhodamine 6G (mitochondrial ATPase inhibitor) led to marked depletion of the intracellular ATP pool [31]. After incubation for 5, 10, 15, 20, and 30 min, samples were centrifugated at 8000× *g* for 2 min. Accumulation of rhodamine 6G was measured as a decline in the fluorescence of the supernatants. Fluorescence was detected by a Varioscan Flash spectrofluorimeter (Thermo Fisher Scientific, Waltham, MA, USA). The excitation wavelength was 515 nm and the emission wavelength was 555 nm.

Active efflux of rhodamine 6G was determined as described previously [32]. Briefly, cells from overnight cultures were incubated in 100 mL of YPD medium and grown to 1 × 10^7^ cells/mL at 150 rpm, 30 °C. The cells were pelleted and washed with sterile deionized water and HEPES buffer (50 mM, pH 7.0). The cells were resuspended in HEPES buffer (50 mM HEPES, pH 7.0) containing 2 mM 2-deoxyglucose and 10 µM rhodamine 6G and shaken for 2 h at 150 rpm, 30 °C. The cells were then washed two times with HEPES buffer (50 mM, pH 7.0) and resuspended in the same buffer. To initiate rhodamine 6G efflux, glucose was added at a final concentration of 2 mM. Samples without glucose were used as the control. After incubation for 5, 10, 15, 20, and 30 min, the samples were centrifugated at 10,000× *g* for 1 min. The rhodamine 6G fluorescence of 100 µL supernatants was measured at the excitation wavelength of 515 nm and the emission wavelength of 555 nm using a Varioscan Flash spectrofluorimeter (Thermo Fisher Scientific, Waltham, MA, USA).

### 2.4. Quantitative PCR

The relative levels of gene expressions were assessed by quantitative PCR (qPCR). Yeast cells were grown in YPD medium until they reached the mid-logarithmic phase. Total RNA was isolated by the hot acidic phenol extraction method as described previously [33]. Then, 1 µg of RNA was used as a template for first-strand cDNA synthesis using Revert AidTM H Minus MMuLV Reverse Transcriptase (Thermo Fisher Scientific, USA). Synthesized cDNA was stored at −20 °C or used immediately in qPCR. The oligonucleotides used in this study are listed in Table S1.

qPCR was prepared using the HOT FIREPol^®^ EvaGreen^®^ qPCR Mix Plus (ROX), 5× (Solis Biodyne, Tartu, Estonia). A thermal protocol for 2-step qPCR (40 cycles of denaturation at 95 °C for 15 s and annealing at 59 °C for 1 min) was set up. Amplification was carried out in the 7900 HT Fast Real-Time PCR System (Applied Biosystems, Foster City, CA, USA). Melting curves obtained after completion of qPCR cycles were used to verify the presence of specific amplicons (95 °C 15 s, 59 °C 15 s, 95 °C 15 s). The reporter signals were analyzed using the ABI SDS 2.2.2 software (Applied Biosystems, USA). *CgACT1* and *CgRDN5.8* were used as reference genes. The Ct values are listed in Table S2. The gene expression levels were determined according to Livak and Schmittgen [34]. Relative changes in expression of the *CgPDR1*, *CgCDR1*, *CgCDR2*, *CgSNQ2*, and *CgPDR12* genes in the parental strain and the *Cgerg6Δ* deletion mutant were compared. Any expression value ≥2 was considered to be indicative of upregulation. All experiments were performed in three parallel wells and three independent replicas. Values are represented as means with SD. For statistical analyses, Student’s *t*-test was used. A *p* < 0.05 (*) was considered statistically significant; *p* < 0.01 (**) highly significant; *p* < 0.001 (***) extremely significant; *p* < 0.0001 (****) highly extremely significant.

### 2.5. Determination of Cell Surface Hydrophobicity

Yeast cells grown overnight in YPD medium were harvested and washed twice with phosphate-buffered saline (PBS) and resuspended in YPD broth to OD_570_ of 0.6–0.8. Cell surface hydrophobicity (CSH) was measured by the water-octane two-phase assay [35]. OD_570_ values of strains in YPD broth without octane overlay were used as negative controls. CSH was calculated as follows: (OD_570_ of the control—OD_570_ after octane overlay)/OD_570_ of the control × 100. For statistical analyses, Student’s *t*-test was used.

### 2.6. Biofilm Formation

The biofilm was prepared according to the protocol of Li et al. [36]. A large loop of cells stored on YPD agar with 2% D-glucose was transferred to YPD broth containing 2% D-glucose and incubated 20 h at 30 °C under agitation at 150 rpm. The cells were then centrifuged at 3000× *g* (Universal 32 R Hettich Zentrifugen, Germany) for 5 min at 15 °C and the pellets were washed twice in PBS. The yeast cell concentration was determined using a hemocytometer. Then, 100 µL samples of the cell suspensions (1 × 10^6^ cells/mL in YPD broth) were placed in 96-well polystyrene flat-bottom plates (Sarstedt, Germany) and the plates were incubated at 30 °C. After 24 h, the non-adherent cells were removed in two washing steps with 200 μL PBS.

Biofilm formation was quantified using the XTT (2,3-bis(2-methoxy-4-nitro-5-sulphophenyl)-2H-tetrazolium-5-carboxanilide; Sigma-Aldrich, St. Louis, MO, USA) reduction test and 0.1% crystal violet staining (Loba Feinchemie, Fischamend, Austria) according to Li et al. [36]. Wells containing the YPD broth were used as negative controls. In brief, the XTT reduction assay composed of 100 μL XTT salt solution (1 mg/mL) and menadione solution (1 μM, Sigma-Aldrich, USA; prepared in acetone) was added to each well containing mature biofilms. The cultures were then incubated for 5 h at 30 °C in the dark. The biofilm cells reduced XTT tetrazolium salt to XTT formazan by mitochondrial dehydrogenases, resulting in a colorimetric change. The amount of color change was measured using a microplate reader at OD490 (Dynex MRX-TC Revelation, Chantilly, VA, USA). Microtiter wells containing only YPD broth without the yeast cells were used as negative controls. In the crystal violet staining method, 100 μL of 0.1% crystal violet was added to each well and the plate was incubated at 30 °C for 20 min. Subsequently, 200 μL of 96% ethanol was added to dissolve the stained biofilm cells and 100 μL of each mixture was transferred to a new 96-well microplate. The absorbance for each well was determined using a microplate reader at OD570 (Dynex MRX-TC Revelation, Chantilly, VA, USA). Similarly, wells containing only YPD broth but not the cells were used as negative controls. For statistical analyses, Student’s *t*-test was used.

### 2.7. Galleria Mellonella Infection

The virulence of *C. glabrata* strains was analyzed in vivo using a *Galleria mellonella* model. *G. mellonella* offers several advantages as a model for studying fungal infections and host–pathogens interactions: I. No ethical approval is needed. II. This model is inexpensive, as this species breeds in large numbers. III. The larvae are easy to handle, meaning that no specific equipment is required IV. This species has a short life cycle, with rapid reproduction. V. Incubation is possible at a range of temperatures. VI. This species’ immune system shares similarities with the mammalian innate immune system. Overnight *C. glabrata* cultures grown in YPD were washed twice with PBS and adjusted to 5 × 10^8^ cells/mL. Three independent groups of 16 randomly chosen larvae were infected with 10 μL of yeast cultures via the last left proleg with a precision syringe (Hamilton microliter syringes, cemented-in needle, Type 701N cap. 10 μL). The needle and the site of injection were disinfected using ethanol. PBS-injected larvae were used as negative control groups to monitor the trauma of the injection (n = 16). The larvae were incubated at 30 °C for 10 days. Survival of larvae was monitored every 24 h by visual inspection. Melanized and non-motile larvae were considered to be dead and removed from the experiment. *C. glabrata* virulence was determined as percentual change in the survival of infected larvae compared to the control group (PBS-injected). For statistical analyses, Fisher’s exact test was used.

## 3. Results

### 3.1. CgERG6 Deletion Confers Increased Susceptibility to Metabolic Inhibitors and Abrogates the Efflux Pump Activity

**Figure 1:**
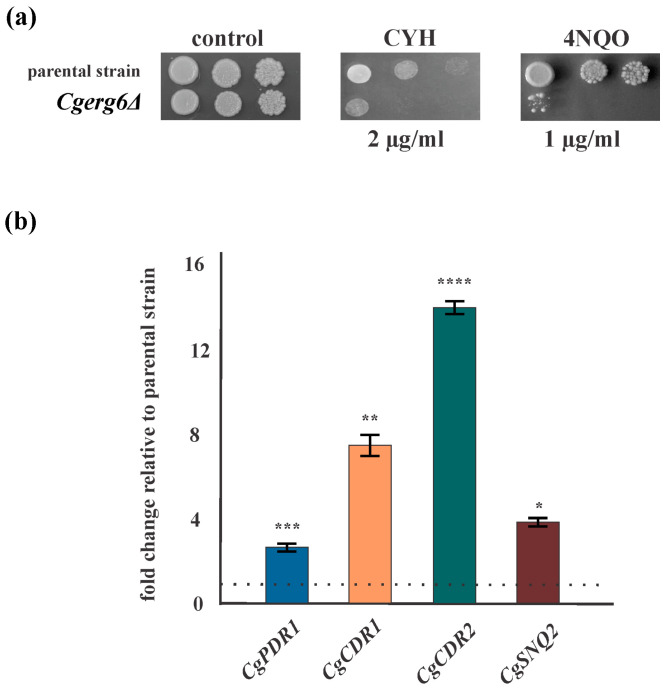
(**a**) Growth of parental strain and *Cgerg6Δ* mutant in the presence of metabolic inhibitors, in which 5 µL aliquots of tenfold serial dilutions (10^7^, 10^6^, 10^5^ cells/mL) of overnight cultures were spotted onto YPD plates and incubated at 30 °C for 2 days. (**b**) Relative gene expression levels of *CgPDR1*, *CgCDR1*, *CgCDR2*, and *CgSNQ2* in *C. glabrata Cgerg6Δ* deletion mutant. The gene transcript level in the parental strain was set as 1. The results are expressed as mean values of three independent experiments (±standard deviation) normalized to the *CgRDN5.8* gene expression level. *p* < 0.05 (*) was considered statistically significant; *p* < 0.01 (**) highly significant; *p* < 0.001 (***) extremely significant; *p* < 0.0001 (****) highly extremely significant.

The *ERG6* gene encodes sterol C24-methyltransferase, which is involved in the late steps of ergosterol biosynthesis. In addition to the previously reported increased tolerance to antifungal azoles and polyenes [27], loss of the *CgERG6* gene resulted in increased sensitivity to cycloheximide and 4-nitroquinoline-N-oxide (4-NQO), compared to cells of the isogenic parental strain (Figure 1a). Cycloheximide, an inhibitor of cytoplasmic protein synthesis, is a substrate of the main efflux pump *Cg*Cdr1p. The mutagen 4-NQO is a substrate for the efflux pump Snq2p. Despite the enhanced susceptibility to metabolic inhibitors in the *Cgerg6Δ* deletion mutant, we observed increased expression of genes encoding the main *C. glabrata* efflux transporters—*CgCDR1*, *CgCDR2*, *CgSNQ2* as well as the gene encoding their transcriptional activator, *CgPDR1* (Figure 1b).

**Figure 2:**
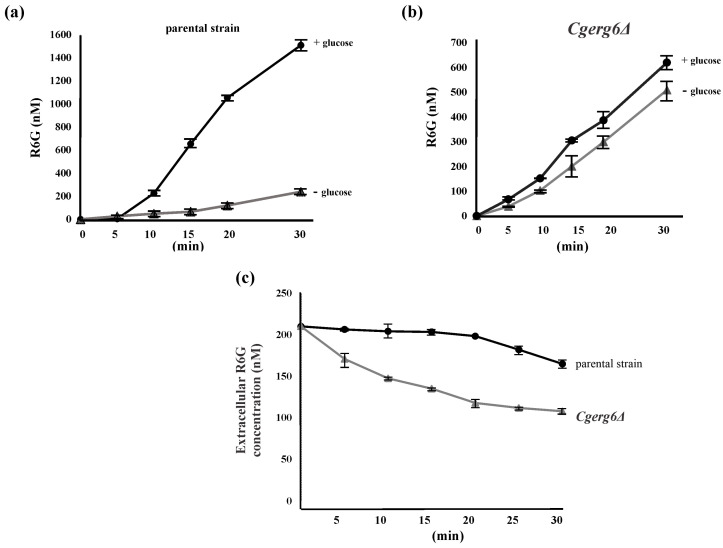
Energy-dependent rhodamine 6G efflux from (**a**) *C. glabrata* parental strain and (**b**) *C. glabrata Cgerg6Δ* mutant. Glucose (final concentration, 2 mM) was added at time zero. (**c**) Rhodamine 6G uptake by the cells of *C. glabrata* parental strain and mutant *Cgerg6Δ* strain. The data represent the mean ± SD of three independent experiments.

In the next experiment, we analyzed the *Cg*Cdr1p transporter efflux activity using the transporter-specific fluorescent dye rhodamine 6G. To analyze the influence of *CgERG6* gene deletion on the function of the Cdr1p efflux pump of *C. glabrata*, we compared the kinetics of rhodamine 6G efflux and accumulation in the *Cgerg6Δ* mutant strain and its isogenic parental strain (Figure 2a–c). The glucose-induced efflux of rhodamine 6G was clearly visible in the parental strain (Figure 2a). However, the addition of glucose to deenergized cells of *Cgerg6Δ* mutant had no effect (Figure 2b). The cells of the *Cgerg6Δ* mutant with abrogated efflux of rhodamine 6G showed higher susceptibility to rhodamine 6G (Table 1). To test whether the difference in passive drug transport is the reason for the increased rhodamine 6G susceptibility of the *Cgerg6Δ* mutant strain, we analyzed the uptake of rhodamine 6G into the cells of both strains. As Figure 2c shows, the *Cgerg6Δ* mutant strain exhibited higher rhodamine 6G accumulation than the parental strain of *C. glabrata*. This observation suggests that the observed susceptibility of the *Cgerg6Δ* strain could be due to increased passive rhodamine 6G uptake into the cells of the mutant.

### 3.2. CgErg6p Is Required for Weak Acid Tolerance in C. glabrata

**Figure 3:**
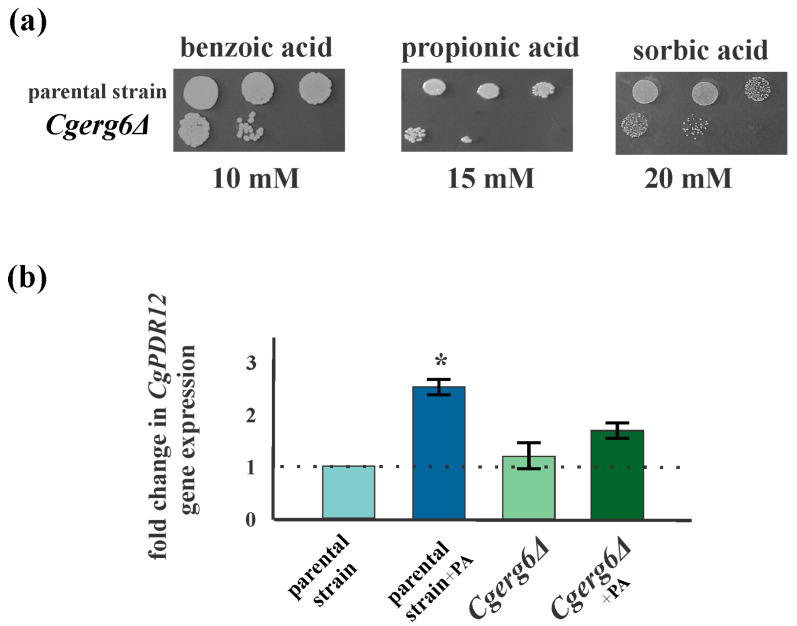
(**a**) Growth of *C. glabrata* parental strain and *Cgerg6Δ* deletion strain in the presence of weak acids, where 5 µL aliquots of cells were spotted as 10-fold dilution (10^7^, 10^6^, 10^5^ cells/mL) on YPD plates containing the indicated concentration of weak acid and grown 2 days at 30 °C. (**b**) Relative gene expression levels of *CgPDR12* gene in parental and *Cgerg6Δ* mutant strains. The gene transcript level in non-treated parental strains was set as 1. +PA cells preincubated for 1 h in the presence of 1 mM propionic acid. The results represent the mean values of three independent experiments (±standard deviation) normalized by the *CgACT1* gene expression level. * *p* < 0.05.

Ergosterol is vital in ensuring stability and plasma membrane integrity in yeast cells. With the loss of plasma membrane integrity, there is an increase in cell permeability to small metabolites, resulting in an increased passive diffusion. Elimination of *Cg*Erg6p increased the susceptibility of *C. glabrata* to weak organic acids that enter into the mutant cells by passive diffusion. The weak organic acids used in our experiments are important food preservatives and powerful fungistatic agents [37]. As Figure 3a shows, the growth of *Cgerg6Δ* mutant strain is reduced in the presence of propionic, sorbic, or benzoic acid as compared to the parental strain. The specific plasma membrane ABC transporter Pdr12p is involved in the active efflux of propionate, sorbate, and benzoate anions from yeast cells. As Figure 3b shows, transcription of the *CgPDR12* gene in the parental strain is induced in the presence of propionic acid. However, the presence of propionic acid in the mutant *Cgerg6Δ* strain did not induce *CgPDR12* gene expression (Figure 3b).

### 3.3. Cell Surface Hydrophobicity and Biofilm Formation

**Figure 4:**
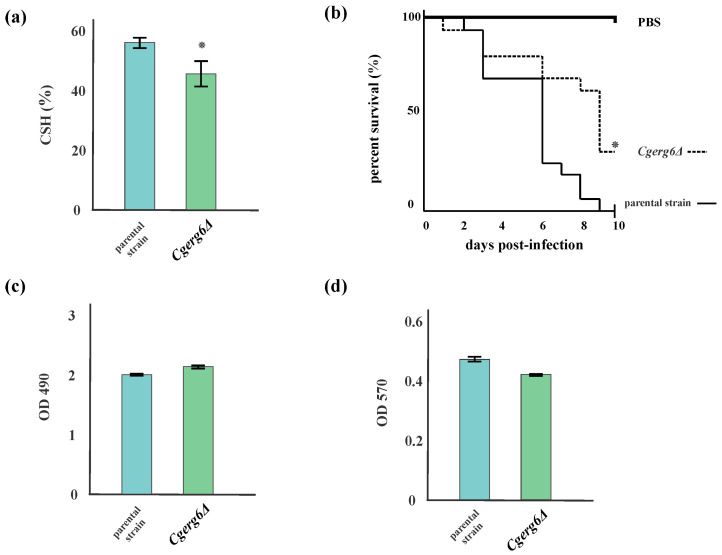
(**a**) Cell surface hydrophobicity (CSH) of *C. glabrata* parental and *Cgerg6Δ* mutant strain. The results represent the means of eight independent experiments (±standard deviation). * *p* < 0.05 (**b**) Survival of *G. mellonella* larvae monitored 10 days after inoculation with parental strain or *Cgerg6Δ* strain. The data represent the result of three independent experiments: *p* < 0.05 (*). (**c**) Biofilm production measured by XXT reduction assay and (**d**) crystal violet staining. The results represent the means of three independent experiments (±standard deviation).

In addition to the overexpression of *CgPDR1* and its target genes, cell surface hydrophobicity (CSH) and biofilm formation are well-recognized virulence factors in *C. glabrata*. They influence cell adherence to biotic and abiotic surfaces, colonization, and pathogenesis of fungal infections. As shown in Figure 4a, the *Cgerg6Δ* mutant strain displayed significantly lower CSH compared to the parental strain. Two assays for quantification of biofilm formation, an XTT (tetrazolium salt reduction) assay (Figure 4c) and a crystal violet staining (Figure 4d) assay were performed. As Figure 4c,d show, the biofilm forming abilities of both the parental and the *Cgerg6Δ* mutant strains were nearly identical.

### 3.4. CgErg6p Contributes to C. glabrata Virulence in a Wax Moth Model

In this experiment, we used *G. mellonella* larvae as a non-vertebrate host model to determine differences in the virulence potential of our *C. glabrata* strains. *G. mellonella* larvae were injected with the *C. glabrata* parental strain and the *Cgerg6Δ* deletion mutant. After infection with *C. glabrata*, the larvae developed a brown-black coloration, indicating that melanin accumulated as part of the insects´ innate immune system. Negative control groups had the lowest level of mortality, activity, and melanization compared to all other groups tested. The survival of the larvae was monitored over time following injection of the yeast suspension, as shown in Figure 4b. As Figure 4b shows, the *Cgerg6Δ* mutant strain was attenuated in virulence compared to that of the isogenic parental strain, resulting in 25–30% of larvae surviving after 9 days at the tested dose.

## 4. Discussion

Eukaryotic membranes are dynamic structures containing many lipid species that contribute to membrane integrity and maintenance. In addition, plasma membrane lipids are important regulators of fungal pathogenicity. Lipids contribute to drug resistance, biofilm formation, and the release of extracellular vesicles. Lipids also influence the mechanical properties of the plasma membrane through the formation of packed microdomains, which are mainly composed of sphingolipids and sterols [38]. Sterols are indispensable molecules for the proper functioning and structural organization of membranes. The chemical structure and concentration of sterols determine the membrane properties and play a central role in regulating the permeability barrier. The change in sterol composition also affects the localization and activity of membrane-bound proteins. The *ERG6* gene encodes a methyltransferase that rearranges the side chain of a fungal sterol. Methylation of the principal membrane sterol at C24 generates the C28 methyl group specific to ergosterol, which represents one of the few structural differences between fungal ergosterol and mammalian cholesterol [39,40].

In this work, we tested the behavior of *C. glabrata erg6Δ* mutant strain in the presence of cycloheximide, 4-NQO, and weak organic acids, and evaluated whether the absence of *Cg*Erg6p influences its virulence potential. We observed increased susceptibility of *Cgerg6Δ* mutant strain to both cycloheximide and 4-NQO. Increased transcription of *CgPDR1* and its target genes did not rescue the ability of the *Cgerg6Δ* mutant to grow in the presence of the tested compounds. Recent reports showed not only the coregulation of *ERG* and drug resistance genes in *C. glabrata*, but also the *Cg*Pdr1p activation by ergosterol depletion [41,42]. The accumulation of ergosterol intermediates could also trigger activation of the ABC transporter-encoding genes to prevent any toxic effects resulting from buildup of ergosterol biosynthetic intermediates. The localization of membrane proteins in the plasma membrane microdomains is a proposed mechanism that could explain the effect of membrane lipids on fungal virulence. Pasrija et al. [43] have shown that the main ABC efflux pump in *C. albicans* (*Ca*Cdr1p) is selectively recruited to plasma membrane rafts for proper localization and functioning. Any imbalance in the raft lipid constituents (sterols or sphingolipids) results in *Ca*Cdr1p sorting errors and loss of function. We propose that the same mechanism occurs in *C. glabrata* that lack Erg6p, leading to a loss of *Cg*Cdr1p transporter efflux activity. The ergosterol concentration in the plasma membrane is also essential to counteract permeabilization induced by weak acid stress [44]. Several specific transporters have been linked to yeast tolerance to various weak acids, including Pdr12p, a plasma membrane ABC transporter involved in the active efflux of propionate, sorbate, and benzoate anions [45,46]. The inability of the *Cgerg6Δ* mutant to tolerate weak acids stress could therefore be the result of non-functioning *Cg*Pdr12p. Guan et al. [47] have shown that modifications in sterol–sphingolipid complexes in the plasma membrane lead to decreases in the activity of *S. cerevisiae Sc*Pdr12p. Our results show that incubation of the *Cgerg6Δ* mutant in the presence of propionic acid did not induce *CgPDR12* gene expression. In contrast, the presence of propionic acid in the parental strain induced the expression of *CgPDR12* almost twofold compared to non-induced conditions. In *S. cerevisiae* weak acid stress causes activation of many genes via the transcription factors Msn2/4p. As Jandric et al. [37] have shown, the *C. glabrata* homologues CgMsn2/4p are apparently not activated by weak acids. In contrast, the *Cg*Hog pathway is activated. The *PDR12* gene was the highest induced gene in both species, and *CgPDR12* required *Cg*Hog1p for full expression [37]. We cannot exclude the possibility that *Cg*Hog1p signaling is impaired in our *Cgerg6Δ* mutant strain. In *S. cerevisiae*, the ABC transporter *Sc*Pdr18p has been proposed to transport ergosterol at the plasma membrane, contributing to the maintenance of adequate ergosterol content and the reduction in stress-induced membrane disorganization and permeabilization. *ScPDR18* is a paralog of *ScSNQ2*, first described as a determinant of resistance to the chemical mutagen 4-NQO [48]. *C. glabrata* has previously diverged to the duplication event in evolution and contains only one *PDR18/SNQ2* homolog. The reported experimental results show a significant overlap between the roles of *Sc*Snq2p and *Cg*Snq2p in multidrug resistance as well as some overlap between the function of *Sc*Pdr18p and *Cg*Snq2p [39]. The increased susceptibility of the *Cgerg6Δ* mutant to weak acids in spite of the increased expression of the *CgSNQ2* gene corroborates the results of Godinho et al. [44], who showed that expression of the *CgSNQ2* gene in *C. glabrata* does not confer protection against stress from weak acids, herbicides, alcohols, or the polyamine putrescine.

The participation of *Cg*Erg6p in ergosterol biosynthesis could explain the observed decrease in cell surface hydrophobicity in the *Cgerg6Δ* mutant cells. These observations point to certain differences in cell wall structure, possibly in terms of the amount or availability of adhesins in our mutant strain, as has been reported for *C. albicans* [49,50]. The cell surface hydrophobicity affects the adhesion, invasive properties, and virulence of *Candida* spp. [49]. In addition to cell wall proteins, changes in cell surface hydrophobicity have also been associated with changes in lipid composition [50,51]. Previous studies have clearly shown that defects in various ergosterol genes affect the virulence of *C. albicans* in murine models [52,53,54]. In this work, we used *G. mellonella* as an in vivo model to study the pathogenicity of the *Cgerg6Δ* mutant strain. *G. mellonella* larvae have been adopted as a simple and inexpensive model for assessing *Candida* virulence [55]. We were able to show that a defect in ergosterol biosynthesis in our *Cgerg6Δ* strain leads to reduced virulence of *C. glabrata* in the *G. mellonella* experimental model. Upon phagocytosis, fungal cells must survive in the harsh environment characterized by carbon source limitation, production of reactive oxygen and nitrogen species and the acidification of the phagosomal compartment [56]. We propose that that the reduced survival of the *Cgerg6Δ* strain may be the result of its increased susceptibility to environmental stresses.

Several inhibitors of methyltransferase activity have been developed and tested, including the transition state analogs (azasterols) or mechanism-based inhibitors (26,27-dehydrozymsoterol). However, off-target toxicity has been observed with azasterol and epiminolanosterol. A promising small molecule working as an allosteric inhibitor with apparently minimal toxicity against *C. albicans ERG6* was tested. These results emphasize the need for future work to find molecules that selectively inhibit Erg6p activity in order to find a novel therapeutic approach for treating invasive fungal infections [57].

## 5. Conclusions

In summary, our findings suggest that *CgERG6* is a critical determinant of virulence and drug resistance in *C. glabrata*, presenting a potential target for novel antifungal therapies.

## Figures and Tables

**Table 1:** Rhodamine 6G inhibits the growth of *Cgerg6Δ* mutant. The data represent the mean ± SD of three biologically independent experiments.

*C. glabrata*	Rhodamine 6G	
	5 mM	20 mM
parental strain	0 ± 0 mm	0 ± 0 mm
*Cgerg6Δ*	7 ± 1 mm	10.3 ± 1.5 mm

The disk diameter is 5 mm.

## Data Availability

The original contributions presented in the study are included in the article/Supplementary Material, further inquiries can be directed to the corresponding authors.

## Supplementary Materials

The following supporting information can be downloaded at https://www.mdpi.com/article/10.3390/jof10100669/s1, Table S1: List of oligonucleotides. Table S2: Ct values.
